# Sex and gender analysis in knowledge translation interventions: challenges and solutions

**DOI:** 10.1186/s12961-020-00625-6

**Published:** 2020-09-23

**Authors:** Amédé Gogovor, Tatyana Mollayeva, Cole Etherington, Angela Colantonio, France Légaré, on behalf of the GIKT Group, Lionel Adisso, Sylvain Boet, Andreea Brabete, Angela Colantonio, Cole Etherington, Amédé Gogovor, Lorraine Greaves, Marie Laberge, France Légaré, Karen Messing, Tatyana Mollayeva, Sylvie-Marianne Rhugenda, Kathryn Sibley, Cora Siebert, Sharon Straus, Dominique Tanguay, Cara Tannenbaum, Cathy Vaillancourt, Krystle van Hoof

**Affiliations:** 1 Vitam - Centre de recherche en santé durable, Pavillon Landry-Poulin, 2525, Chemin de la Canardière, Québec, G1J 0A4 Canada; 2 Tier 1 Canada Research Chair in Shared Decision Making and Knowledge Translation, Université Laval, Quebec, Canada; 3 Health and Social Services Systems, Knowledge Translation and Implementation Component of the Quebec SPOR-SUPPORT Unit, Quebec, Canada; 4 Department of Family Medicine and Emergency Medicine, Université Laval, Quebec, Canada; 5 Rehabilitation Sciences Institute, Faculty of Medicine, University of Toronto, 160-500 University Avenue, Toronto, M5G 1V7 Canada; 6 Toronto Rehabilitation Institute-University Health Network, University of Toronto, 160-500 University Avenue, Toronto, M5G 1V7 Canada; 7 Clinical Epidemiology Program, Ottawa Hospital Research Institute, 501 Smyth Road, Rm L1287, Ottawa, ON K1H 8L6 Canada

**Keywords:** Gender, sex, healthcare, knowledge translation, research design, intersectionality

## Abstract

Sex and gender considerations are understood as essential components of knowledge translation in the design, implementation and reporting of interventions. Integrating sex and gender ensures more relevant evidence for translating into the real world. Canada offers specific funding opportunities for knowledge translation projects that integrate sex and gender. This Commentary reflects on the challenges and solutions for integrating sex and gender encountered in six funded knowledge translation projects. In 2018, six research teams funded by the Canadian Institutes of Health Research’s Institute of Gender and Health met in Ottawa to discuss these challenges and solutions. Eighteen participants, including researchers, healthcare professionals, trainees and members of the Institute of Gender and Health, were divided into two groups. Two authors conducted qualitative coding and thematic analysis of the material discussed. Six themes emerged, namely Consensus building, Guidance, Design and outcomes effectiveness, Searches and recruitment, Data access and collection, and Intersection with other determinants of health. Solutions included educating stakeholders on the use of sex and gender concepts, triangulating perspectives of researchers and end-users, and participating in organisations and committees to influence policies and practices. Unresolved challenges included difficulty integrating sex and gender considerations with principles of patient-oriented research, a lack of validated measurement tools for gender, and a paucity of experts in intersectionality. We discuss our findings in the light of observations of similar initiatives elsewhere to inform the further progress of integrating sex and gender into the knowledge translation of health services research findings.

## Introduction

For almost two decades, the Canadian Institutes of Health Research (CIHR) have been leaders in funding knowledge translation (KT) research, known elsewhere as implementation science. Coined by CIHR, KT is defined as a dynamic and iterative process that includes synthesis, dissemination, exchange and ethically sound application of knowledge to improve the health of Canadians, provide more effective health services and products and strengthen the health care system [1]. This application of research knowledge can involve patients, clinicians, public health officials, health service managers and policy-makers [2, 3]. KT interventions facilitate the uptake of research evidence into practice and/or policy, i.e. they are interventions that make use of research results [3]. KT interventions can include patient and clinician education programmes, evidence-based guidelines, policy-making, promotion of self-management or continuous quality improvement programmes.

However, it is now becoming clear that KT must take sex and gender into consideration, as these are critical equity issues for intervention uptake in areas such as decision-making and communication as well as for stakeholder engagement, needs and interests [4]. A KT strategy may function differently and has different impacts depending on the sex and gender identity of researchers, participants and end-users as well as on the gender relations among them. Theories and frameworks operate differently within and across sexes and genders. There is growing evidence too that applying a sex and gender perspective enhances the accuracy and relevance of findings. Moreover, if sex and gender are not considered, KT programmes can reproduce or even increase existing inequities [4, 5].

CIHR has been a leading institution in advancing sex and gender considerations in health research. Starting in 2013, the CIHR Institute of Gender and Health (IGH) initiated a strategic plan with a central focus on sex, gender and health to advance the integration of sex and gender considerations throughout all phases of the health research cycle [6]. For the purposes of this paper, we define sex and gender based on the CIHR definitions, as follows: sex refers to a set of biological attributes in humans and animals while gender refers to socially constructed roles, behaviours, expressions and identities of girls, women, boys, men and gender-diverse people [7]. Sex and gender interact and affect different aspects of social roles, relations, norms and identity [8–10]. White women and black women, for example, may share determining factors for health based on sex but other determining factors, such as race and ethnicity or class, may differ substantially [11]. Intersectionality is an approach to understanding and analysing how these different factors operate and influence each other in the organisation of power in a given society [12, 13]. The intersectional lens is used in many studies that address sex and gender, including three out of the six KT projects we discuss [14–16].

We aimed to reflect on challenges and solutions found in integrating sex- and gender-based analysis (SGBA) into six KT interventions.

## The funded projects

Six research teams were funded by the CIHR IGH to (1) generate evidence about whether applying SGBA to KT interventions involving human participants improves effectiveness; (2) contribute to a broader knowledge base on how to integrate sex and gender into KT interventions; and (3) facilitate the consideration and development of gender-transformative approaches (i.e. approaches that promote gender equity) in KT interventions [6, 17, 18]. The call echoed several international recommendations, including the Madrid Statement, urging WHO and the member states to include sex-disaggregated information in their data collection systems and develop gender-sensitive indicators to achieve the highest standard of health and support effective health policy [19, 20]. These six ongoing projects (Box 1) are taking place in the context of teaching hospitals, community-based health and social centres, workplaces, professional organisations, and KT developers.

## Conduct of the meeting and synthesis of the discussions

Eighteen participants (15 women and 3 men; 8 French-speaking) including 9 researchers, 3 healthcare professionals, 4 trainees, 1 CIHR IGH staff member, and Dr. Cara Tannenbaum, Scientific Director of the CIHR IGH (cihr-irsc.gc.ca/e/12735.html), attended the meeting (Additional file 1 for agenda).

Prior to the meeting, team leaders were asked to identify three challenges they had met within the course of their project for which strategies were found and three challenges for which solutions had not yet been found. These lists of challenges with and without solutions were shared among teams ahead of the meeting along with recent relevant publications to give each team member time to consider ideas and prepare suggestions. Participants were divided into two discussion groups and, after the discussion, reported back to the larger group. Subsequently, each team revised or updated their pre-meeting texts based on the discussions. Afterwards, two authors (AG, TM) conducted a thematic analysis of the revised pre-meeting texts, the summaries reported back to the larger group, and notes taken by the team members during the discussions [21]. The two authors independently conducted inductive initial coding of the texts with iterative discussions for consensus throughout the process. Themes were reviewed by other members of the teams, including team members who were not present at the meeting (Additional file 2).

Verbal consent was obtained from the participants regarding further use of the output of the meeting. We received a waiver from the Centre intégré universitaire de santé et de services sociaux de la Capitale-Nationale Ethics Board.

## Challenges of integrating sex and gender into KT

For some challenges, teams found solutions that fully resolved the problem. However, for others, there were issues for which none of the teams felt they had found adequate solutions. Challenges, solutions and unresolved challenges were organised into six themes and are presented following the phases of a KT intervention, namely Consensus building, Guidance, Design and outcomes effectiveness, Searches and recruitment, Data access and collection, and Intersection with other determinants of health.

### Consensus building

Due to the interdisciplinary nature of sex and gender research as well as the importance of stakeholder involvement in KT, research teams could become quite large and unwieldy, with widely varying perspectives and levels of engagement. The teams found it difficult uniting around a common vision and a common language on sex and gender. For example, there was disagreement within research teams on defining sex and gender concepts (e.g. in the CPD team), on whether to treat sex and gender as non-binary variables and on whether there is enough evidence to include sex and gender considerations in clinical guidelines (e.g. in the TBI team).

*Solutions*: Better communication, i.e. sharing of material and a development of a consensus on terms and concepts before the implementation process begins, regular team meetings, monthly newsletters, and regular working sessions.

*Unresolved*: For the CPD team, developing a research process that (1) combines the world of sex and gender experts with that of KT experts, (2) uses a patient-oriented research lens and (3) is committed to patient engagement, is a complex and as yet unresolved challenge. Another challenge still unresolved was successfully communicating the complexity and novelty of the topic of sex and gender to research partners. Another unresolved issue was research partners who do not engage and how to take them into account in the knowledge translation process.

### Guidance

Teams found there was a lack of guidance regarding integrating concepts of sex and gender into models, frameworks or guidelines. For example, the OR team had difficulty integrating sex and gender considerations into the Theoretical Domains Framework (TDF), and the TBI team found a lack of theories to explain sex and gender influences in TBI.

*Solutions*: Regarding incorporating gender into the TDF, the OR team sought feedback from multiple co-investigators with expertise in sex- and gender-based analysis. The team proposed that the TDF could be used as a coding structure only and that, rather than posing the traditional TDF questions, questions could incorporate the project context and be more free-flowing in style. The TBI team believed that, for the moment, the best approach was less direct, i.e. to capture sex and gender effects in TBI through the unique voices and narratives of women and men with TBI, their significant others, and clinicians.

### Design and effectiveness outcomes

#### Study designs

The methodological and ethical relevance of using study designs with control groups in studies initiated at the behest of or in partnership with sex or gender subgroups was questioned. Is it ethically sound or relevant to reproduce the intervention with another subgroup not mobilised in the same way by the same question? Another challenge is taking gender into consideration (as opposed to sex) in studies with small cohorts without losing statistical power and, finally, how to integrate gender into data analysis, for example, in studies on how firefighters’ work or pregnant women’s work affects their health (environmental/occupational health team).

The CPD team, who conducted a controlled trial, found limited evidence for adding sex- and gender-based content to their experimental CPD training on diabetes and depression. It also struggled with managing the new experimental training: it required more time than the control training that excluded it and risked diluting core health-related content. The control also risked reaffirming existing sex or gender biases in the original training. The same team, for example, noted that the intervention group noticed and appreciated sex and gender notions while the control group noticed (and complained about) the absence of sex and gender notions.

*Solutions*: Present more social factors in the control training. The CPD team added a qualitative dimension to the intervention in the form of an interactive component (following the theoretical component) including a brainstorming activity and a panel discussion on stigmatisation related to depression and/or diabetes, medication, and gender.

#### Other design issues

If investigators are not aware of their own biases or of power differentials, design methods risk re-naturalising or reaffirming sex or gender biases. There may be a power disequilibrium between researchers and partners or between partner A and partner B, resulting in some voices being louder than others in the KT process. As the environmental/occupational health team asked, are methods such as Delphi, for example (concept definition based on a consensus approach), valid in the presence of social groups that do not have the same power and voice strength?

*Solutions:* Identify methods that will produce valid and authentic data from excluded or less empowered populations. For example, the occupational/environmental health team advised 'don’t mix men and women in the same focus group if you suspect a risk of power imbalance'. Give strength to the voices of the most excluded in dissemination initiatives.

#### Effectiveness outcomes

Identifying outcomes for evaluating community-based KT interventions that integrate sex and gender was a challenge for all teams. Which criteria should be used to determine its success or effectiveness? Effectiveness, as evaluated qualitatively by researchers versus by end-users or stakeholders, can be different, depending on their understanding or experience of sex and gender, and can complicate the dissemination of research results. In addition, the scientific community and academic journals are conservative about evaluation models and often closed to experimental ones.

*Solution*: Instead of using the controlled trial study design, the occupational/environmental health team conducted retrospective analyses, triangulated the perspectives of researchers and users, and focused on process and intermediate indicators by describing different interventions (case studies) rather than seeking to compare interventions that cannot be compared. Involve stakeholders in the co-construction and validation process, then triangulate views when interpreting and disseminating results. Findings should be interpreted and disseminated with a consciousness of sex and gender bias or stereotypes. For example, as one team noted, if injury risk factors are different for women and men, do not presume women are weaker. Therefore, it is important to carry out strategic and innovative initiatives to share knowledge with a broader audience, such as being on journal editorial boards, being peer reviewers, influencing journal ratings by selecting the best ones in regard to sex/gender considerations, and refusing to publish in journals that do not accept papers with innovative methods or topics.

### Searches and recruitment

#### Searches

Adding sex and gender to search terms in certain KT domains can unearth a huge and unmanageable volume and variety of returns. The substance use team, whose systematic review questions were multi-component (with four substances and three levels of intervention), had over 22,000 returns on their search.

*Solutions:* The substance use team narrowed the research question, modelled searches based on key papers and developed an SGBA categorisation for the prioritisation of papers.

#### Recruitment

It can be challenging to recruit participants who identify themselves as gender diverse to capture a complete spectrum of sex and gender data, risking their exclusion from interventions that could benefit them. For example, the TBI team were unable to recruit anyone who self-identified as gender diverse.

*Solutions:* Connect with relevant organisations and develop trusting relationships with them. For example, the TBI team and the occupational/environmental health team contacted organisations with ties to the LGBTQ+ (Lesbian, gay, bisexual, transgender, queer, and other diverse options) community and created partnerships with them.

### Data access and data collection

#### Usable data

Baseline data, common definitions of terms, and so-called cleansed data (where incorrect, incomplete, improperly formatted, or duplicated data are amended or removed) are required metrics against which implemented changes can be measured. Context-specific data in a usable format (i.e. disaggregated) was not always available for these teams, for example, usable data relating to environmental and occupational health, on depression, and on diabetes. When data are presented by sex or gender, often, little attention is paid to explanatory hypotheses and mechanisms. Furthermore, in the literature, the terms “sex” and ‘gender” are frequently used interchangeably. The substance use team, who were conducting a systematic review, found that the term ‘gender” is particularly misunderstood, often being interpreted as gender identity, as sex, or as referring to women only.

*Solutions*: Participation in organisations and publishing to influence policy and practice, giving lectures and conferences to relevant organisations. For example, the occupational/environmental health team founded the International Ergonomics Association Technical Committee on Gender and Work to lobby for more sex- and gender-sensitive investigations and interventions, organised and edited a special issue of the journal *Applied Ergonomics* dealing with gender issues in ergonomics, and gave lectures in universities and labour organisations to explain why sex- and gender-sensitive analysis contributes to better interventions.

#### Data collection

Teams struggled with a lack of understanding of the meaning of sex and gender among participants, regardless of whether they were patients, caregivers or clinicians. This lack of understanding could result in participants being alienated or offended by questions on sex and gender. The TBI team, for example, found that participants who were asked directly about sex and gender, especially those with experience of TBI, often confused the terms, conflated them with sexuality, or declined to answer the questions. In addition, some were unwilling to be educated on the topic.

*Solutions*: Although the TBI team used CIHR definitions to explain the terms, they explained that TBI frequently affects socially marginalised populations and found that, rather than asking direct questions, it is through asking about how patients’ lives had been impacted by their injuries and hearing their stories that they come to understand their “roles, behaviors, and expectations” and gain a deeper understanding of participants’ sex and gender constructs. The occupational/environmental health team suggested using fact sheets with short, illustrative and shocking examples of how sex or gender bias can influence research results and practice and getting to know the point of view of stakeholders and other researchers before trying to educate them.

### The intersection of sex and gender with other determinants of health

The intersectional team was confronted with the issue of how to ensure sex and gender are not diluted when considering their intersection with the multiple other determinants of health and the exclusion of other important determinants such as Indigeneity.

*Solutions*: The intersectional team found that the methods for striking a balanced representation of sex and gender considerations were capacity-building, a consensus process with all representative stakeholders, including in-person and online discussions, and finally voting on which frameworks and tools to use.

*Unresolved:* Paucity of experts in intersectionality, difficulty in operationalisation of intersectionality, and lack of researchers’ experience in KT.

## Discussion

We reported the discussions of a meeting that was held in November 2018 in Ottawa to examine challenges CIHR-funded team members have faced and to share key strategies. Scientific methods to study sex and gender in health research are still evolving, with limited agreement between and within stakeholder groups on knowledge and education needs on the concept of sex and gender.

Although some of the challenges are relevant to sex- and gender-sensitive research per se, others are specific to KT. These challenges are related to educating (sometimes unwilling) researchers and participants about sex and gender, understanding of terms, the unwieldy nature of large integrated KT projects that involve many disciplines and stakeholders, and integrating constructs of sex and gender into educational material for healthcare professionals and patients in the presence of limited scientific evidence on the topic.

The lack of guidance for integrating sex and gender into KT interventions made it difficult for these researchers to operationalise the construct, as did the confusion or disagreements over the terms sex and gender [22]*.* The concept of gender is particularly complex and was a major source of confusion [23]. While sex and gender are now recognised as non-binary variables and researchers are urged to move beyond binary conceptualisation of sex/gender [22, 24], with no methodological advice or evidence-based recommendations to guide them, it is still challenging for researchers to grapple with sex- and gender-based analysis in KT.

The challenges can occur at every stage and involve researchers (e.g. lack of theoretical guidance), patient experts and other stakeholders (e.g. disagreement on terms) or participants (e.g. recruitment). Different study designs had different challenges – for example, challenges to do with control interventions were relevant to trials, challenges with literature searches were especially relevant to a systematic review and challenges to do with power imbalances could particularly affect a Delphi consensus study.

The teams reported challenges pertaining to difficulties in recruiting gender-diverse participants and in assessing or collecting disaggregated data. These difficulties are compounded by the use of inconsistent terminology in the literature, difficulty in applying the concepts, a failure to come to terms with the impact of sex and gender on research design and outcomes, and challenges with data collection and datasets. These findings have been confirmed in a review of literature on sex- and gender-based analysis [25]. While progress has been made in this respect in some research fields, such social science health research [22, 26], implementation literature reveals that attention to sex and gender has not yet infiltrated research methods in the field of KT [4]. The strategies proposed by the teams may begin to fill this gap.

Another challenge was the difficulty in operationalisation of intersectionality. Various international bodies, including the WHO, have recognised the importance of intersectional research [8, 27]. Recent calls for research strategies for both higher and low- and middle-income countries (LMICs) recommend sex and gender analysis as an entry point into a deeper intersectional analysis [27, 28]. Yet methods for integrating intersectionality into research and policy are still in their very early stages of development.

The problems of effectiveness outcomes found by the teams are interesting to compare with those found by ADVANCE teams (similar initiatives in the United States) and Athena SWAN teams (in the United Kingdom). The contribution of ADVANCE to increasing inclusivity has been difficult to separate from overall institutional and academic pushes for inclusivity; additionally, their projects include small numbers of individuals and multiple interventions, which makes it difficult to isolate change [11]. The Athena SWAN project also found it difficult to directly link the effects of policy changes with their initiatives [29].

Finally, these difficulties or challenges experienced by the teams, both those with solutions and those that remain unresolved, share one striking element when considered as a whole – they are all struggling to deal with complexity. The difficulty of operationalising intersectionality in KT studies is related to the complexity of considering so many determining factors together. The difficulty of theorising or developing models or guidelines on sex and gender considerations is related to the fact that they are fluid and deeply complex notions. The difficulty of using controlled trial design in integrating sex and gender into KT relates to the complexity of assessing their components and determining any meaningful outcome. The difficulties of managing large multidisciplinary KT teams that include sex and gender experts as well as patients and other stakeholders is related to the complexity of managing a vast array of perspectives and representing everyone’s interests equally. This is confirmed in a study comparing the Athena SWAN award scheme with other gender equality initiatives in Europe, which found that interventions are complex social interventions in a complex system [29]. A multitude of contextual variables relate in complex, non-linear ways and must constantly adapt to moving targets and new conditions. Studies that include sex and gender considerations and other intersecting factors may be complex but are a more inclusive and therefore faithful representation (or measure) of reality. Yet, the irony is that the knowledge that KT studies are expanding and disseminating is ultimately produced by the scientific method, which is often more about excluding rather than including. It is unsurprising then that the scientific community may not always welcome this complexity because it calls into question the paradigm of the scientific method itself [30]. Further research is needed on the obstacles that prevent investigators from taking account of gender and sex, on papers that have influenced investigators in a positive way and how this happened, and on best forums for promoting discussion of these issues.

The paper is subject to several limitations. The meeting was part of the CIHR Impact of Gender on KT Interventions grant and, as such, it was not initially planned for data collection and analysis; in addition, the discourse of team members present at the discussion may not have been representative of the key researchers in each project. Second, the meeting was held in a high-income country with a resource-intensive health system; thus, the findings may not represent all the challenges in this respect, although a few participants were from LMICs and one of the teams had a project in a LMIC (Table 1). While a study on child development that covered 41 LMICs found that few major gender differences emerged [31], supporting a gender similarities view, the integration of sex and gender into KT interventions would vary widely depending on institutional and cultural norms. Finally, there were no patient participants at the meeting, although some teams included patient partners.

**Table 1:** Description of the Institute of Gender and Health funded projects

Project	Objective of research project	Setting/target population	Study design	Sex/gender analysis
Does One Size Fit All? The Implications of Gender for Operating Room (OR) Team Performance, Teamwork Interventions, and Equitable Patient Outcomes (Team 1)	(1) To examine the interrelationship between OR team gender composition, individual clinician sex for each professional role, intraoperative teamwork performance and patient outcomes; (2) to qualitatively explore barriers and facilitators to effective teamwork in the OR according to clinicians’ gender and professional role using the Theoretical Domains Framework (TDF)	Two academic hospitals in Ontario, Canada Core OR team members (scrub and circulating nurses, anaesthesiologists, surgeons, residents, fellows)	(1) Prospective observational design with quantitative analysis; (2) qualitative interviews following the TDF	Explicit examination of individual sex, group gender norms, sex/gender differences; disaggregation of data
A gender transformative approach to improve outcomes and equity among persons with Traumatic Brain Injury (Team 2)	(1) Document important concepts and ideas for education on topics of sex and gender in the TBI context; (2) develop educational materials for patients with TBI, significant others and clinicians providing care that accounts for sex/gender; and (3) test the application of these educational materials for feasibility and effectiveness	Rehabilitation research-teaching hospital/adult patients with TBI; significant others; clinicians working with patients with TBI	Qualitative interviews Evidence synthesis Educational intervention, cluster-randomised trial in KT	Sex and gender analysis (taking into account biological sex, age, marital status, education and ethnicity)
Integrating and measuring the effect of sex, gender and gender transformative approaches to substance use treatment, prevention and harm reduction in Canada [16] (Team 3)	To systematically review evidence on sex- and gender-related factors affecting the use and response to four substances – alcohol, nicotine, cannabis and opioids; to interview leaders and to survey Knowledge Attitudes and Practice (KAP) among workers in three pilot settings; to co-develop three interventions regarding sex and gender and substance use and responses and re-assess KAP among workers in three pilot sites	The substance use system via three pilot sites – (1) a territorial government addictions workers training programme; (2) a provincial addictions foundation that provides training, programming and information; and (3) a family service treatment centre and its aftercare system partners in a Canadian province	Systematic review of co-development of interventions in three pilot sites; before and after study of KAP before and after introducing sex and gender into three different aspects of the substance use system in Canada: Nunuvat addictions worker training course; Manitoba Addiction Foundation women’s treatment and Saskatchewan aftercare system components	Mixed methods: compilation of sex- and gender-related factors and evidence that affect substance use for women, men, and gender and sexual minorities; use of evidence to underpin the co-development of three pilot interventions in aspects of the substance use system
Modelling an approach to gender-conscious participatory action-oriented research and knowledge transfer favouring equality, equity and occupational/environmental health [32] (Team 4)	Interventions to improve the inclusion of both sexes/genders in environmental and occupational health efforts by unions and other community groups Incorporation of sex-gender based analysis in occupational/environmental health interventions by graduate students who have been trained to do so	Workers facing health challenges; communities in the Amazon region affected by toxic exposures Graduate students in occupational (ergonomics) and environmental health	Multiple case studies based on interviews with researchers and community representatives; search for correlates of successful and unsuccessful incorporation of sex/gender considerations; mixed methods	Description of contexts, methods and impacts of intervention studies that have considered sex/gender to some extent in the process Comparison of successful and unsuccessful interventions
mATriCES-F: ApplicaTion des Connaissances axée sur le gEnre et le Sexe des personnes en contexte Francophone [Sex- and gender-oriented knowledge translation in Francophone contexts] (Team 5)	Increase sex- and gender-sensitive clinical behaviours and attitudes among French-speaking healthcare professionals in Canada through continuing professional development (CPD) activities	CPD developers, clinicians, patients and other CPD stakeholders from francophone communities in Ontario, Quebec and New Brunswick	Interventional non-randomised controlled trial	Data will be disaggregated by sex and will include sex-specific analysis
Transforming the practice of KT: Embedding gender (Team 6)	The objective of this study is to encourage knowledge translation (KT) intervention developers who are addressing the needs of older adults to use an intersectional approach when designing and implementing KT interventions; our goal is to develop and evaluate intersectionality-enhanced versions of KT frameworks and associated tools for three prioritised stages of KT	KT intervention developers from across Canada	Mixed methods	Data will be analysed by gender

## Conclusions

These findings provide a sampling of the challenges and solutions found in implementing KT projects that consider sex and gender. They have implications for every stage of the KT process, from identifying the knowledge to be translated to disseminating it in an equitable way. Integrating sex and gender in KT is a new field and these findings will illuminate the path ahead.

Box 1 Settings and goals of the six Institute of Gender and Health-funded knowledge translation research projects (further details in Table 1)

**Table:** 

Team 1, based in Ontario, Canada, is investigating the impact of gender on the effectiveness of teamwork in the operating room with clinicians in urban academic hospitals (**‘the OR team’**).	
Team 2, based in Ontario, Canada, is documenting concepts and ideas for developing and testing educational materials on sex and gender for traumatic brain injury (TBI) care (**‘the TBI team’**).	
Team 3, is performing a systematic review on evidence on sex and gender factors affecting substance use (alcohol, cannabis, nicotine and opioids) and is using the evidence in three pilot interventions on substance use in Manitoba, Nunavut and Saskatchewan, Canada (**‘the substance use team’**).	
Team 4 seeks to model a sex- and gender-conscious participatory action approach in occupational health and environmental health interventions by unions and other community groups in the Amazon and in Quebec, Canada (**‘the occupational/environmental health team’**).	
Team 5 aims to increase sex- and gender-sensitive clinical behaviours and attitudes through continuing professional development (CPD) activities in French-speaking urban and rural communities in Quebec, New Brunswick and Ontario, Canada (**‘the CPD team’**).	
Team 6, a pan-Canadian team, aims to develop and evaluate intersectional approaches to KT frameworks and associated tools with KT intervention developers working with older adults (**‘the intersectional team’**).

## Supplementary information

**Additional file 1.** Agenda of the impact of gender on KT interventions team grant recipient meeting.

**Additional file 2.** List of team representatives.

## Data Availability

The text files used and/or analysed during the current study are available from the corresponding author on reasonable request.

## Abbreviations

CIHR: Canadian Institutes of Health Research
CPD: continuing professional development
IGH: Institute of Gender and Health
KT: knowledge translation
LMICs: low- and middle-income countries
TBI: traumatic brain injury
TDF: Theoretical Domains Framework
