# Supplementary material for: Sex and gender analysis in knowledge translation interventions: challenges and solutions
Source: Health Res Policy Syst. 2020 Sep 23;18:108. doi: 10.1186/s12961-020-00625-6 (PMC7509920; doi:10.1186/s12961-020-00625-6)
Supplement: Supplementary file 1 — Additional file 1. Agenda of the impact of gender on KT interventions team grant recipient meeting. [file 12961_2020_625_MOESM1_ESM.docx]

Additional file 1. Agenda of the impact of gender on KT interventions team grant recipient meeting

Impact of Gender on Knowledge Translation Interventions Team Grant Recipient Meeting

Agenda

Date: November 23, 2018

Location: The Ottawa Hospital, Civic Campus (1053 Carling Avenue) – Kaminski Room

Meeting Objectives:

1. Discuss challenges teams have encountered within their research projects and

brainstorm solutions.

2. Facilitate networking and knowledge exchange among interdisciplinary research teams.

3. Support continued leadership in research on gender and health within Canada.

| 8:00-9:00am | Breakfast |
| --- | --- |
| 9:00-9:05am | Welcome and Introductions  • *Dr. Sylvain Boet (PI), Scientist, Clinical Epidemiology Program, Ottawa Hospital Research Institute; Associate Professor, Department of Anesthesiology & Pain Medicine, University of Ottawa*  • *Dr. Cole Etherington (Sex & Gender Champion), Research Associate, Clinical Epidemiology Program, Ottawa Hospital Research Institute* |
| 9:05-9:25am | Canadian Institutes of Health Research update  • *Dr. Cara Tannenbaum, Scientific Director, CIHR Institute of Gender and Health* |
| 9:25-10:45am | Presenting challenges and solutions (small groups)  • Teams will be divided in small groups to discuss the challenges they have encountered so far and solutions they have found |
| 10:45-11:00am | Break |
| 11:00am-12:00pm | Brainstorming solutions for remaining challenges (small groups)  • Teams will discuss potential solutions to the challenges they have not yet found solutions for within their small groups |
| 12:00-12:45pm | Lunch |
| 12:35-1:45pm | Challenges and solutions (large group)  • Each small group will summarize the challenges and solutions they discussed for the larger group |
| 1:45-2:15pm | Keynote  • *Dr. Ivy Bourgeault, CIHR Chair in Gender, Work and Health; Professor, Telfer School of Management and Institute of Population Health, University of Ottawa* |
| 2:15-2:30pm | Closing |
