# Supplementary material for: Sex and gender analysis in knowledge translation interventions: challenges and solutions
Source: Health Res Policy Syst. 2020 Sep 23;18:108. doi: 10.1186/s12961-020-00625-6 (PMC7509920; doi:10.1186/s12961-020-00625-6)
Supplement: Supplementary file 2 — Additional file 2. List of team representatives. [file 12961_2020_625_MOESM2_ESM.docx]

Additional file 2. List of team representatives

| Name | Role | Affiliation |
| --- | --- | --- |
| Lionel Adisso | PhD candidate, Epidemiology  Lead CIHR IGH Trainee Network | Faculty of Medicine, Université Laval |
| Sylvain Boet | Associate Professor; Scientist | Department of Anesthesiology and Pain Medicine & Department of Innovation in Medical Education, University of Ottawa;  Ottawa Hospital Research Institute |
| Andreea Brabete | Research Associate, Women’s Health | Department of Personality, Centre of Excellence for Women's Health, University of British Columbia |
| Angela Colantonio | Professor and Director,  Acquired Brain Injury Team Leader | Rehabilitation Sciences Institute, University of Toronto; Toronto Rehabilitation Institute-University Health Network |
| Cole Etherington | Senior Research Associate  Sex/gender Champion | Clinical Epidemiology Program, Ottawa Hospital Research Institute |
| Amédé Gogovor | Postdoctoral Research Fellow  Co-lead CIHR IGH Trainee Network | Faculty of Medicine, Université Laval |
| Lorraine Greaves | Senior investigator, Clinical Professor | Centre of Excellence for Women's Health, University of British Columbia |
| Marie Laberge | Associate Professor | **School of Rehabilitation**, Université de Montréal |
| France Légaré | Professor of Family Medicine and Clinician  Member, IGH Institute Advisory Board | Faculty of Medicine, Université Laval;  Tier 1 Canada Research Chair in Shared Decision Making and Knowledge Translation |
| Karen Messing | Professor Emeritus  Sex/gender Champion | Biological Sciences, Université du Québec à Montréal |
| Tatyana Mollayeva | Associate Director  Sex/gender Champion | Rehabilitation Sciences Institute, University of Toronto |
| Sylvie-Marianne Rhugenda | Research Development Advisor | Faculty of Social Sciences, Université Laval |
| Kathryn Sibley | Associate Professor and Director of Knowledge Translation Platform | Department of Community Health Sciences, University of Manitoba;  George & Fay Yee Centre for Healthcare Innovation |
| Cora Siebert | Communications Specialist | Institute of Gender and Health, Canadian Institutes of Health Research |
| Sharon Straus | Professor of Medicine and Director of Knowledge Translation Program | St. Michael’s Hospital, University of Toronto;  Tier 1 Canada Research Chair in Knowledge Translation and Quality of Care |
| Dominique Tanguay | Research Development Advisor | Faculty of Social Sciences, Université Laval |
| Cara Tannenbaum | Professor and Director | Institute of Gender and Health, Canadian Institutes of Health Research;  Université de Montréal |
| Cathy Vaillancourt | Professor and Director | INSR-Centre Armand Frappier Santé Biothechnologie;  Director, University of Quebec intersectorial health research network |
